# Investigation of Methods to Extract Fetal Electrocardiogram from the Mother’s Abdominal Signal in Practical Scenarios

**DOI:** 10.3390/technologies8020033

**Published:** 2020-06-05

**Authors:** Sadaf Sarafan, Tai Le, Amir Mohammad Naderi, Quoc-Dinh Nguyen, Brandon Tiang-Yu Kuo, Tadesse Ghirmai, Huy-Dung Han, Michael P. H. Lau, Hung Cao

**Affiliations:** 1 Department of Electrical Engineering and Computer Science, University of California, Irvine, CA 92697, USA;; 2 Department of Electronics and Computer Engineering, Hanoi University of Science and Technology, Hanoi 10000, Vietnam;; 3 Division of Engineering and Mathematics, University of Washington, Bothell Campus, Bothell, WA 98011, USA;; 4 Sensoriis, Inc., Edmonds, WA 98026, USA;; 5 Department of Biomedical Engineering, University of California, Irvine, CA 92697, USA

**Keywords:** Fetal ECG extraction, independent component analysis (ICA), extended Kalman filter (EKF), blind source separation (BSS), fetal home monitoring

## Abstract

Monitoring of fetal electrocardiogram (fECG) would provide useful information about fetal wellbeing as well as any abnormal development during pregnancy. Recent advances in flexible electronics and wearable technologies have enabled compact devices to acquire personal physiological signals in the home setting, including those of expectant mothers. However, the high noise level in the daily life renders long-entrenched challenges to extract fECG from the combined fetal/maternal ECG signal recorded in the abdominal area of the mother. Thus, an efficient fECG extraction scheme is a dire need. In this work, we intensively explored various extraction algorithms, including template subtraction (TS), independent component analysis (ICA), and extended Kalman filter (EKF) using the data from the PhysioNet 2013 Challenge. Furthermore, the modified data with Gaussian and motion noise added, mimicking a practical scenario, were utilized to examine the performance of algorithms. Finally, we combined different algorithms together, yielding promising results, with the best performance in the F1 score of 92.61% achieved by an algorithm combining ICA and TS. With the data modified by adding different types of noise, the combination of ICA-TS-ICA showed the highest F1 score of 85.4%. It should be noted that these combined approaches required higher computational complexity, including execution time and allocated memory compared with other methods. Owing to comprehensive examination through various evaluation metrics in different extraction algorithms, this study provides insights into the implementation and operation of state-of-the-art fetal and maternal monitoring systems in the era of mobile health.

## 1. Introduction

Telemedicine and mobile health (m-Health) have been mentioned for more than a decade. However, only recently, have the wearable technology, internet of things (IoTs), computation power as well as telecommunication (going to 5G and beyond) reached a point where these have become possible. Further, in special scenarios such as the current covid-19 pandemic, effective distanced care and monitoring are in need more than ever. However, a feasible approach to prenatal care to help limit exposure to the novel coronavirus is pivotal, even after the pandemic ends.

A recent national study reported by the Centers for Disease Control (CDC) showed that the U.S. fetal mortality rate remained unchanged from 2006 through 2012 at 6.05 per 1000 births [1]. A key fetal monitoring parameter, which is fetal heart rate (fHR) via cardiotocography (CTG), despite being used in 85% of all labors in the U.S., and with comparable frequency during the antepartum period for monitoring, has not unequivocally shown that it can reduce perinatal mortality. The traditional CTG based non-stress test (NST) and contract stress test (CST) for fetal health assessment are all done in the clinic or hospital under the supervision of a healthcare professional. The current CTG uses the Doppler ultrasound method to measure fHR. Such measurement could be challenging at times due to the need for precise alignment with the fetal heart to detect the fHR, which could be difficult when there are excessive maternal or fetal movements, or in the case of maternal obesity [2].

To overcome the fHR measurement difficulties for CTG using the Doppler method, especially needed during a situation of possible fetal distress when fHR assessment is absolutely critical, obstetricians have resorted to measuring fHR by the more reliable method of using fECG, which currently can only be obtained through a scalp electrode directly attached to the fetal scalp [3]. However, this is an invasive procedure and can only be done after the rupture of the amniotic membrane, potentially causing some risks such as infection. Non-invasive fetal electrocardiography (NI-fECG) is among the most promising of the alternative methods for continuous fetal monitoring. It can be achieved by measuring fECG along with maternal electrocardiogram (mECG) using skin-contact electrodes placed on the mother’s abdomen. However, non-invasive acquisition of full-feature fECG from maternal abdominal recordings is not an easy task. The low signal-to-noise-ratio (SNR) of fECG and the appearance of other signals, namely mECG, baseline wander, and noise, bring challenges. A number of reports has been presented towards the development of new signal processing techniques to tackle such issues. Filtering techniques, including adaptive filtering [4–8], Kalman filtering [9–11] and wavelet transform [12,13] are among the popular ones. Although filtering techniques are very effective for single-channel ECG denoising, there are two major limitations of fECG extraction: (1) it requires an additional reference signal for adaptive filtering while a precise QRS complex (the main spike observed in an ECG graph) of the mECG signal is needed for the Kalman filter method; (2) most adaptive filtering-based methods are not robust and fail to extract fECG, particularly when the fECG and mECG signals are temporally overlapped [14]. Blind source separation (BSS) methods with well-known algorithms, including principle component analysis (PCA) and independent component analysis (ICA), have been used for fECG extraction [15–17]. The BSS methods assume that the abdominal electrocardiogram (aECG) is a mixture of independent signals, consisting of fECG, mECG and noise. While it shows promising performance in fECG extraction, the order of the separated independent component could not be determined, thus it is challenging to identify the fECG component for further process [18]. A number of parameters (e.g., *t*-test, correlation coefficient, heart rate) has been used for the automatic identification of the extracted component [19–21]. Template subtraction (TS) is another widely used approach. The method involves subtracting a synthetic mECG, which is generated by estimating the QRS complex waveform (mQRS) of mECG, from the abdomen signal [22–27]. The main challenge of this method involves mQRS detection [28], and it becomes more challenging if the fetal R waves overlap maternal R waves.

Those aforementioned approaches have been successfully applied to extract fECGs from aECGs, and the efficacy of these methods have been carried out by synthetic data in some studies. Nevertheless, working on real data is much more challenging, thus fECG monitoring in daily life cannot be done yet. To address this, various methods, such as Extended Kalman Filter (EKF), template subtraction (TS), independent component analysis (ICA) and their combinations, were rigorously investigated using a set of NI-fECG data in Cardiology Challenge 2013 [29]. Furthermore, we tested the efficacy of these methods with the data modified by adding different types of noise, mimicking practical scenarios that could be encountered in the home setting. A comprehensive performance metric, including F1 score, computational complexity (i.e., execution time and allocated memories), and noise robustness, was used to assess the performance.

## 2. Materials and Methods

The data were taken from the PhysioNet 2013 Challenge databank which consists of a collection of one-minute aECG recordings [30]. Each recording includes four noninvasive abdominal signals collected by electrodes placed on the mother’s abdomen, containing a mixture of both the fetal and maternal ECG signals. The data were obtained from multiple sources using a variety of instrumentation with differing frequency responses, resolution, and configuration. However, in all cases, the sampling rate was presented as 1000 samples/s. In each recording, in addition to four noninvasive abdominal signals, reference QRS complex annotations were manually marked by a team of experts. In this work, we used set A of the dataset, which contains 75 records, excluding a number of recordings (a33, a38, a47, a52, a54, a71, and a74) that had inaccurate reference annotations [31].

### 2.1. Extended Kalman Filter

Bayesian filtering is a probabilistic technique that uses incoming measurements y and a mathematical process model to recurrently estimate the posterior distribution of a hidden state random variable X at each time k [32]. The conventional Kalman Filter (KF) assumes a linear model for the system dynamics and observation equations. In practice, however, most systems are nonlinear in nature. The EKF is an extension of the standard KF to nonlinear systems. A dynamic model of the system may be represented as follows

(1) $$\{\begin{array}{l} {\underline{x}}_{k}=F_{k-1}{\underline{x}}_{k-1}+{\underline{w}}_{k-1}\\ {\underline{y}}_{k}=H_{k}{\underline{x}}_{k}+{\underline{v}}_{k} \end{array}$$

where *F*_*k*−1_ is the state transition model applied to the previous state, *x*_*k*−1_, *w*_*k*_, and *υ*_*k*_ correspond to the process and observation noise, which are assumed to be white, zero-mean, uncorrelated $\left(E\left[{\underline{v}}_{k}{\underline{w}}_{k}^{T}\right]=0\right)$ with associated covariance matric $Q_{X}=E\left\{{\underline{v}}_{k}{\underline{w}}_{k}^{T}\right\}$ and $R_{X}=E\left\{v_{k}w_{k}^{T}\right\}$, respectively [22]. It is further assumed that the components of the noise processes are uncorrelated, i.e., *Q*_*X*_ and *R*_*X*_ are diagonal. *H*_*X*_ is the observation model that maps state space into the observed space. The KF estimates the state *x*_*k*_ based on the knowledge of the system dynamics and the noisy measurements *y*_*k*_ . When a rather precise measurement of the states of a system is valid, the diagonal entries of *R*_*X*_ are small, and the KF gain is adapted so as to rely on that specific measurement. However, for the epochs where data are too noisy or there are no measurements available, the *R*_*X*_ entries are large and the KF tends to follow its internal dynamics rather than tracking the observations.

McSharry et al. developed a dynamic model with a set of three differential equations to generate synthetic ECG signals in Cartesian coordinate system [33]. Further, Sameni et al. transformed the model to a polar coordinate system and provided a convenient discrete-time mathematical model [34]. The model represents an ECG signal by a sum of five Gaussian functions, each function corresponding to the five waves of an ECG signal, P, Q, R, S, T waves. The state vector of the dynamic model is defined by *x*_*k*_ = [*θ*_*k*_, *z*_*k*_]^*T*^, and the state equation is given by

(2) $$\{\begin{array}{l} \theta_{k+1}=\left(\theta_{k}+\omega \delta \right)\;\text{mod}\;2\pi \\ z_{k+1}=-\underset{i\in \left\{P,Q,R,S,T\right\}}{\sum }\frac{\alpha_{i}\Delta \theta_{i}\omega \delta }{b_{i}^{2}}\text{exp}\left(-\frac{\Delta \theta_{i,k}{}^{2}}{2b_{i}^{2}}\right)+z_{k}+\eta_{k} \end{array}$$

where Δ*θ*_*i*,*k*_ = (*θ*_*k*_ – Ψ_*i*_)mod(2*π*) is the phase increment, *δ* is the sampling period, *η*_*k*_ is the state noise, and *α*_*i*_, *b*_*i*_ and *ψ*_*i*_ represent the amplitude, width, and center of the Gaussian functions of the five PQRST waves. The measurement vector is defined by *y*_*k*+1_ = [*ϕ*_*k*+1_, *s*_*k*+1_]^*T*^, where *ϕ*_*k*+1_ is the observed phase representing the linear time wrapping of the R-R time interval into [0, 2*π*], and *s*_*k*+1_ is the observed amplitude. The measurement equation is given by (*u*_*k*+1_ and *v*_*k*+1_ denote the measurement noises).

(3) $$\{\begin{array}{c} \phi_{k+1}=\theta_{k+1}+u_{k+1}\\ s_{k+1}=z_{k+1}+v_{k+1} \end{array}$$

### 2.2. Template Subtraction (TS)

**Figure 1.**
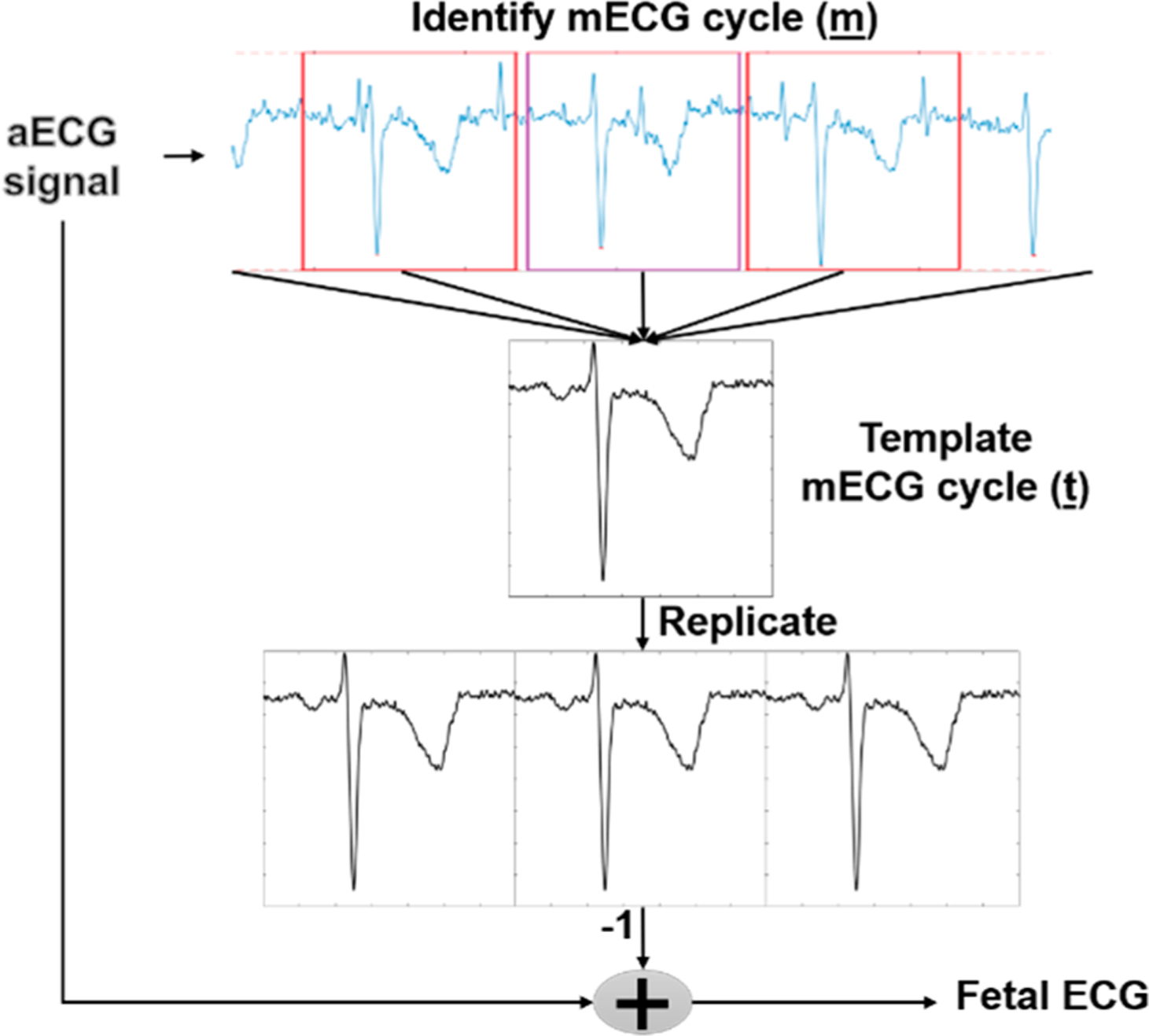
Template subtraction (TS)’s illustration for abdominal electrocardiogram (aECG).

The main idea of TS is to regenerate mECG and then subtracting it from the aECG. Based on maternal QRS detection, we identify each mECG cycle m belonging to 0.25 s before and 0.45 s after maternal R peak positions with respect to the duration of the whole cardiac cycle. The template maternal ECG t then was formed by taking the average of all mECG cycles, and the new mECG was obtained by replicating the t as shown in Figure 1. Another improved method based on the TS was utilized in this work, namely TSc. In this method, the template maternal ECG cycle t was scaled with a constant *α*. The scaling of t reduces the mismatch between the average and the actual mECG cycle m, which is caused by the time-vary morphology of the mECG [35,36]. The scaling constant *a* was based on the search for the least-mean square (LMS) e^2^ error between m and t, as shown in the following formula:

(4) $${\text{e}}^{2}=\text{min}\left\|\underline{t}\text{a}-\underline{m}\right\|$$

### 2.3. Independent Component Analysis (ICA)

ICA is a mathematical technique for recovering unobserved source signals (components) from observed signal mixtures. Let us denote a matrix **X** = [**x**_1_, **x**_2_, **x**_3_, … , **x**_**n**_] considered as observed signals which are assumed to be linear. Instantaneous mixtures of the source signals are denoted by a matrix **S** = [**s**_1_, **s**_2_, **s**_3_, … , **s**_**n**_]. We can present the relationship between **X** and **S** by the following equation:

(5) X=AS

where matrix **A** represents a *n* × *n* mixing matrix and contains the mixture coefficient. The goal of ICA is to find the unmixing matrix **W** (i.e., the inverse of **A**) that will give the matrix **Y**-the best possible approximation of **S** by:

(6) Y=WX≅S

For this purpose, a number of criteria can be considered on the basis of the maximization of non-gaussianity, maximum likelihood and minimization of mutual information, to name a few [18]. Typically, ICA algorithms can be broken into several steps, including centering, whitening and iterative algorithm. While the centering step is utilized to make the signal a zero-mean variable, whitening step is applied for transforming the observed signals so that the new processed observed signals are white (i.e., its components are uncorrelated and their variances equal to unity) [18]. The whitening step is necessary as it can significantly simplify the ICA problem. For iterative algorithm steps, there are number of formulations for this procedure. For instance, JADE algorithm was first developed as an application of blind identification in beamforming [37,38] which is iterative with a defined number of iterations. The FastICA algorithm [18] is considered to be the most popular method among ICAs due to its simplicity, convergence speed and satisfactory results in numerous applications. This algorithm is often used in ‘real time’ applications because of the possible parallel implementation. It converges quickly as it seeks for the components one by one. FastICA uses simplified kurtosis for the independent component estimation, and the detail of this algorithm has been summarized in the **Algorithm 1 chart**. Another optimization algorithm is RobustICA. Compared with FastICA, RobustICA uses a general kurtosis contrast function to maximize the non-gaussianity, as shown in the following **Algorithm 2 chart**. The process of this method is described in [39]. Overall, using RobustICA has some advantages over FastICA, such as (1) pre-processing is not required which allows one to deal with all signal types; and (2) RobustICA uses an adaptive step size, ensuring that the weights converge to the actual convergence point, thus avoiding getting trapped as the former algorithm does.


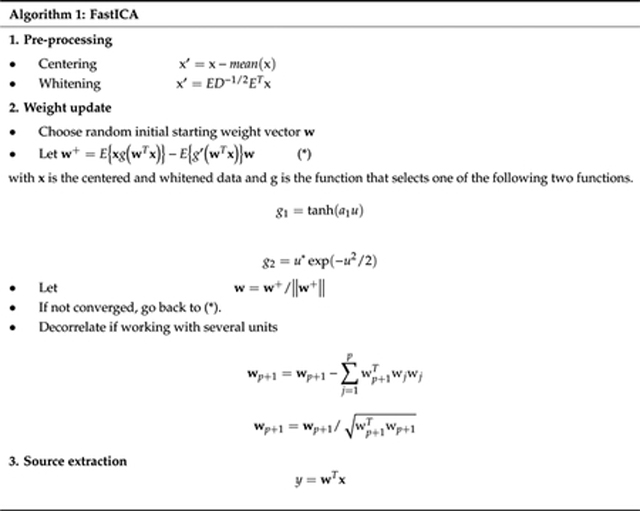



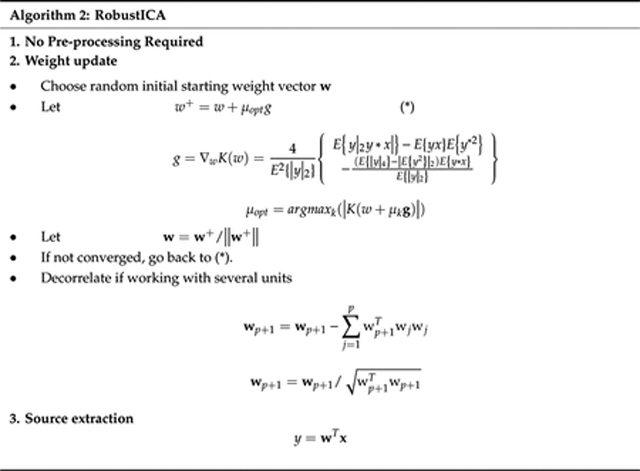


In this work, we explored three algorithms (i.e., JADE, FastICA and RobustICA) for fECG extraction to evaluate their efficacy in terms of accuracy, computational complexity and noise robustness. Furthermore, combining the TS method and ICA method can yield superior performance, as shown in a previous study [32]. Therefore, we attempted to use the combinations as: (1) TS-ICA in which the aECGs were first applied TS method to remove the mECG component, ICA method was then used to extract fECG. This method is useful as mECG’s amplitude is dominant in the aECG, the use of TS may help to retain the fECG component; (2) ICA-TS in which the aECGs were first applied by ICA method, and four new separated signals produced were then put through the TS method. Utilizing ICA at the beginning is for extracting fECG, mECG and other components; however, it does not completely remove mECG in the extracted fECG. Thus, applying the TS method could eliminate the remaining mECG component; and (3) ICA-TS-ICA in which the aECGs went through three steps; specifically, after removing mECG from the residual of (ICA-TS), ICA could be used again. It may have better results because four sources in residual signals have not showed mECG, so with using ICA in the last step, there will be three noise channels and one fECG channel.

### 2.4. Fetal QRS Detection

#### 2.4.1. Fetal QRS Detection with EKF

**Figure 2.**
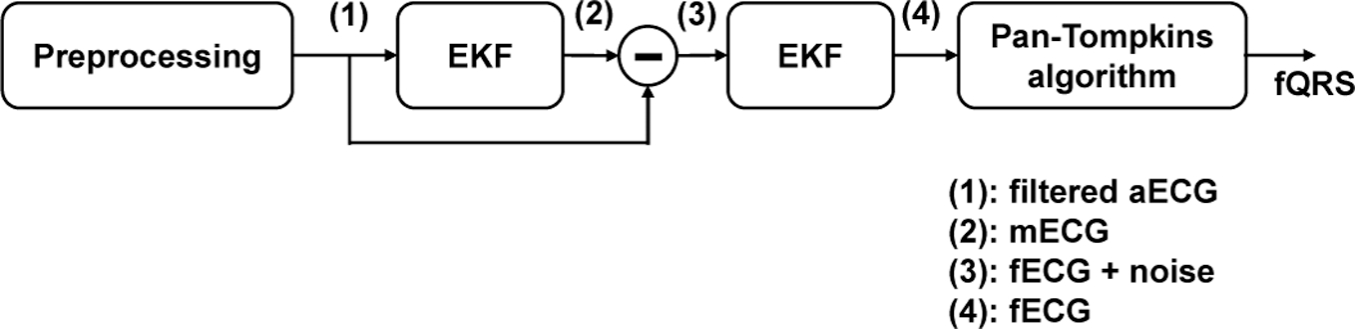
Fetal QRS (fQRS) detection process: (1) Preprocessing step with low pass filter utilized; (2) extended Kalman filter (EKF) applied for maternal ECG (mECG) extraction; (3) mECG subtracted from filtered aECG signal and EKF used for fetal ECG (fECG) extraction; (4) The Pan-Tompkins algorithm applied for fQRS detection.

Based on this dynamic model, several algorithms employing EKF for the extraction of fECG from abdominal signals have been proposed. One such algorithm used sequential EKF algorithms. First, the baseline wander was removed by using a low pass filter, assuming the processed signal is a mixture of the mECG, fECG and noise. Next, EKF was employed to extract mECG. The fallowing step is removing the mECG signal by subtracting it out from the processed signal. The output of this step is fECG along with noise, which another EKF was used to extract the fECG from. Finally, fetal QRS complex (fQRS) was detected using the Pan-Tompkin algorithm, as shown in Figure 2 [34].

#### 2.4.2. Fetal QRS Detection with TS, ICA and Their Combination

**Figure 3.**
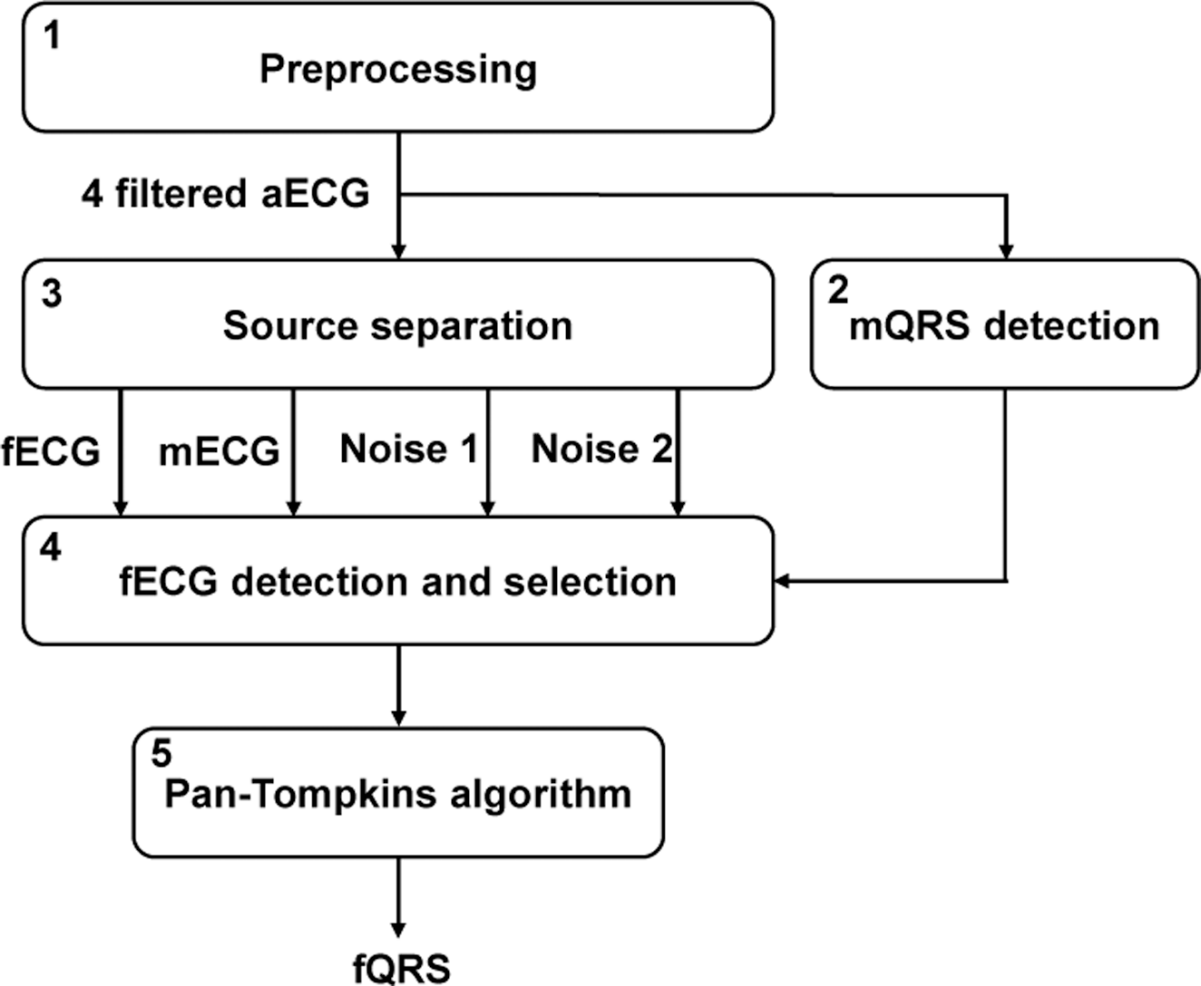
fQRS detection process: (**1**) Preprocessing step with notch filter, high pass filter and low pass filter utilized; (**2**) The Pan-Tompkins algorithms applied for mQRS detection used to create a template mECG and for channel selection in independent component analysis (ICA) method; (**3**) Source separation includes different approaches (TS, ICA and its hybrid). For ICA and the hybrid method, the extracted signals contain 4 signals (i.e., fECG, mECG and two noise signals; (**4**) Using mQRS detection from (**2**) as a criterion for fECG selection; (**5**) The Pan-Tompkins algorithm applied for fQRS detection.

**Figure 4.**
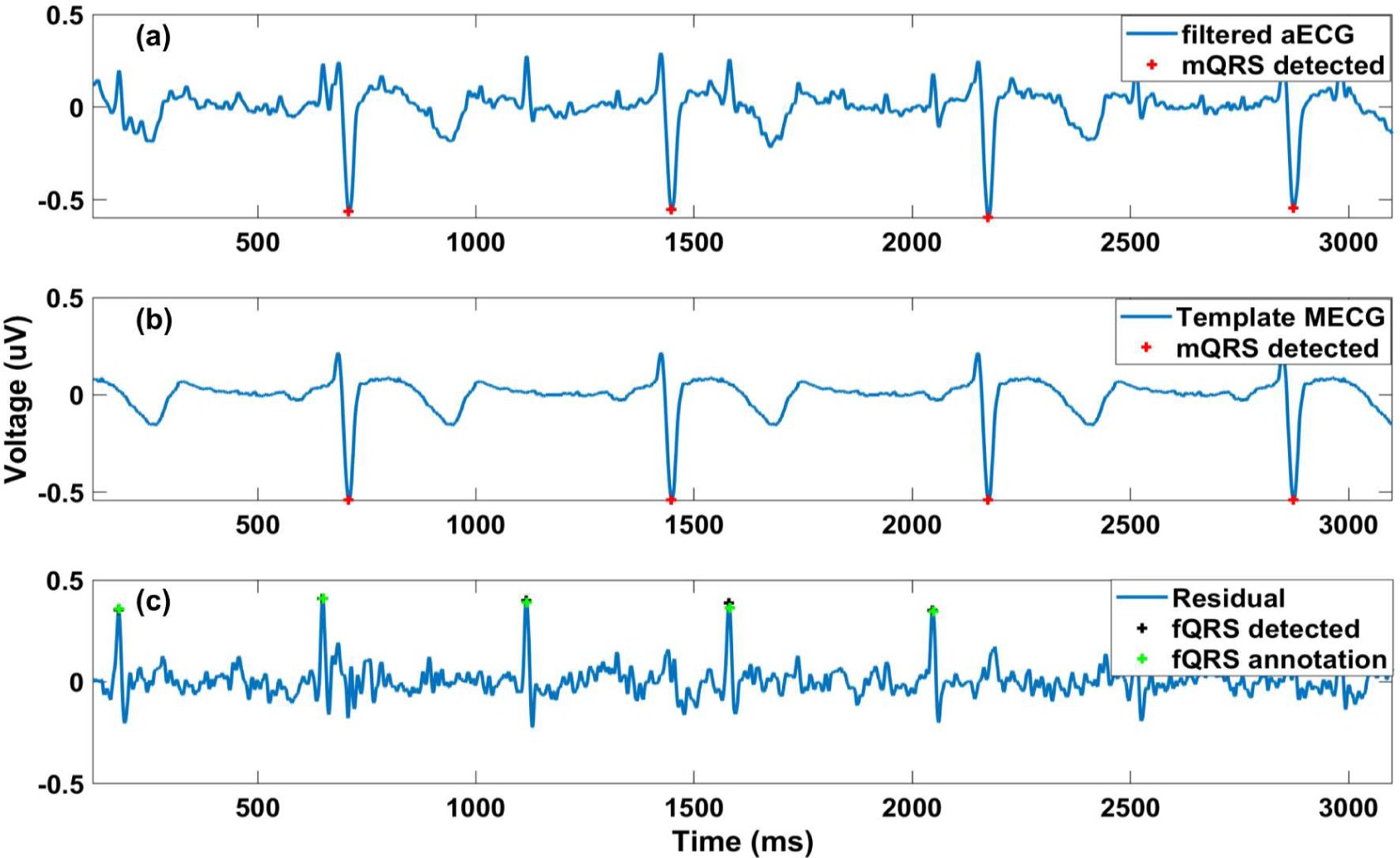
fQRS detection illustrated by TS method: (**a**) the aECG signal is filtered baseline wander and power line and applied Pan-Tompkins for mQRS detection; (**b**) a template of mECG is constructed from filtered aECG and the R peaks of mECG; (**c**): the residual signal is derived by the subtraction between filtered aECG and template mECG and Pan-Tompkins is applied for fQRS detection. The fQRS annotation is also included (plus sign in green).

Figure 3 illustrates the fQRS detection process with TS, ICA and their combinations. First, the aECG signals were preprocessed to remove the baseline wander, power line noise and high-frequency noise. Specifically, a notch filter with the cutoff frequency of 50 Hz was utilized to remove the power line noise while a high pass filter and a low pass filter were deployed to eliminate the baseline wander and high-frequency noise with the cutoff frequency of 10 and 99 Hz, respectively. Second, it should be noted that the mECG’s amplitude is usually larger than other components in aECG. Thus, the Pan-Tompkin algorithm [40] was implemented on four aECG signals, resulting in R peaks of mECG (mQRS detection), as shown in Figure 4a. Third, the algorithms in the source separation block, which could be the TS method, ICA method or combined methods, have been used. In the case of TS, as described in Section 2.2, after mQRS detection, a template mECG was produced (Figure 4b). Subsequently, we subtract the aECG with the template mECG, resulting in four residuals. Finally, the Pan-Tompkin algorithm was used to detect fQRS (Figure 4c). With the available fQRS reference annotations, the channel having the highest F1 score would be chosen. In the case of ICA, three different ICA methods (i.e., JADE, FastICA and RobustICA) were utilized in source separation. After applying ICA, the output would be fECG, mECG and two other noise signals. The reference mQRS would be used to select the mECG channel in the output. The Pan-Tompkin algorithm was then utilized for other channels (i.e., fECG and two other noise signals). Finally, the fECG channel was chosen based on a smoothing indicator (SMI). Specifically, the SMI was defined as the number of occurrences, over each-minute segments, where the absolute value of the change in instantaneous heartrate is more than 29 beats per minute [16]. This threshold was empirically determined on the data and which channels had the lowest SMI, it was referred to fECG channel.

### 2.5. Experiments

#### 2.5.1. Modified Signals with Gaussian Noise Added

The aECG acquired in the daily life would include various kinds of noise. According to the Central Limit Theorem, these tend toward a normal distribution. Therefore, Gaussian noise with different amplifications has been added to the data to mimic the real scenarios. Specifically, the normally distributed random noise was generated by the random function in MATLAB with the lowest amplitude ranging from −4 to 4 μV, which is denoted as noise level 0. Then, different noise levels were achieved by multiplying with constants divisible by 3 (i.e., 3, 6, 9, 12, 15, 18 and 21).

#### 2.5.2. Modified Signals with Motional Artifacts Added

**Figure 5.**
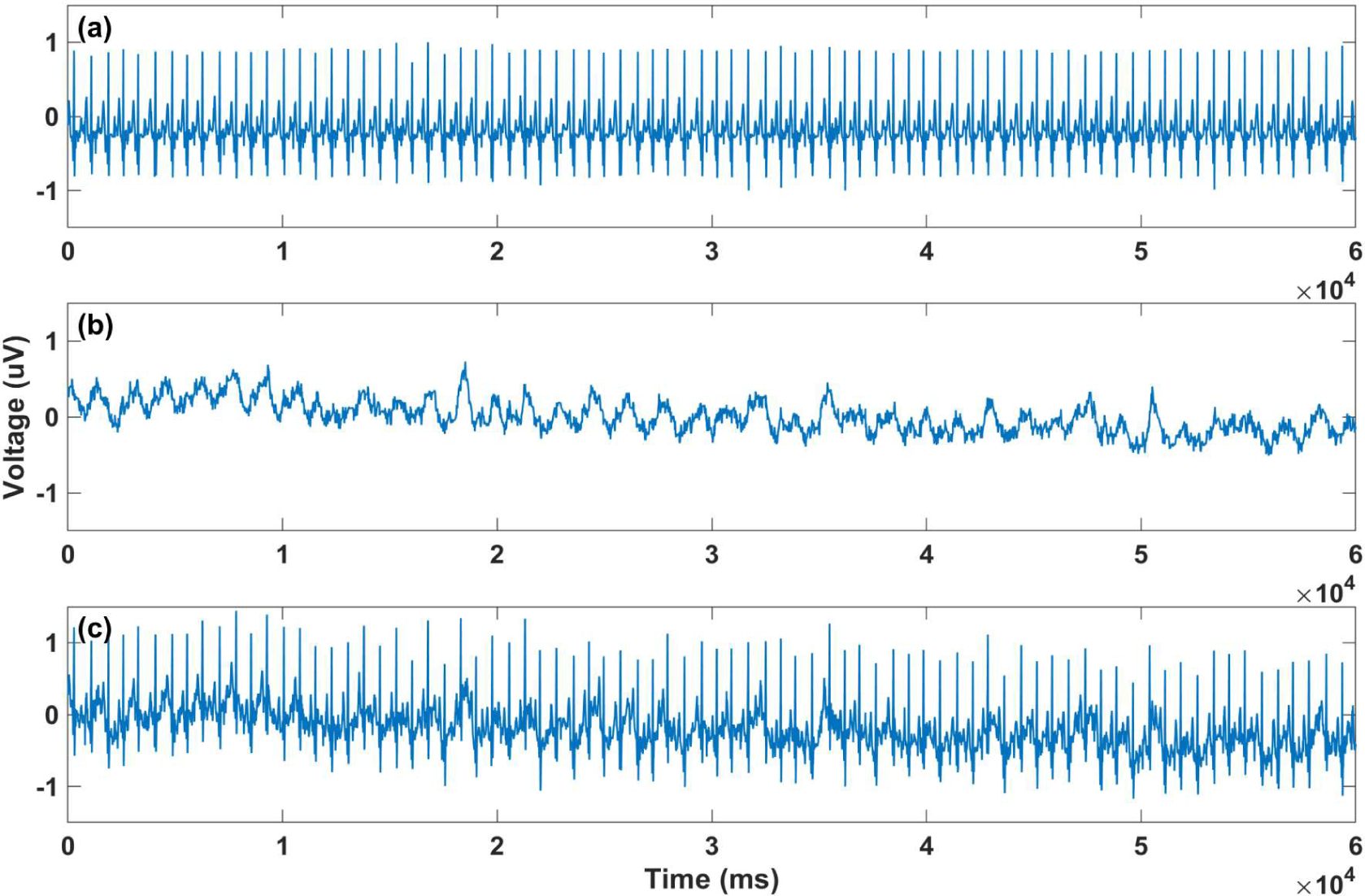
Illustration of applying noise to record a01 with motion added: (**a**) Normalized a01 record; (**b**) Generated motion noise; (**c**) a01 with added motion noise artifact.

The dataset was obtained in the clinical setting, where motion noise was mostly avoided because the subjects were in resting position, so the movement artifacts should be added to the data for practical applications. We attempted to generate a realistic motion noise. First, the ECG data were recorded from a healthy subject during different types of activities such as walking. For the ECG recording, the OpenBCI Cyton board (OpenBCI, Brooklyn, NY, USA) was used with two of its default electrodes. The board communicates wirelessly with a computer. Since the amplitude of the noise is an important factor, normalization had been used for all of signals to reinsure aECG and the motion noise have realistic amplitudes. For extracting motion noise, the acquired data first should be normalized between −1 and 1 and then by using EKF, motion noise and filtered ECG data are achieved. In the second step, for adding the motion noise to aECG, the aECG data should be normalized with the same threshold. In the last step, the motion noise is added to the normalized aECG (Figure 5).

### 2.6. Comparison Schemes

#### 2.6.1. F1 Score

A statistical analysis to assess the accuracy of detected positions of the fQRS extracted by the aforementioned algorithms was performed by comparing with the positions of the annotated fQRS. We computed F1 scores of the proposed algorithms as follows

(7) $$F_{1}=\frac{2\times TP}{2\times TP+FN+FP}$$

where *TP*, *FP* and *FN* are true positive (correctly identified fQRS), false positive (wrongly detected fQRS) and false negative (missed fQRS) detections, respectively. Note that the error of for fQRS detection (*FP*) is evaluated using a window-based metric (i.e., the positions of detected fQRS were wrong if it did not belong to 50 ms before and 50 ms after the positions of the annotated fQRS).

#### 2.6.2. Time Execution

This assessment aims to compare the complexity among methods, hereby providing suggestions to implement the algorithms to other platforms with different computational capacities. Here, a computer with the following configuration was used: Intel Core i5-8400 CPU @ 2.80 GHz 6 Cores; Window 10 Education x64bit; RAM 16GB DDR4 and the software Matlab R2016. The execution time of each algorithm is calculated from the end of pre-processing to the end of R peak detection.

#### 2.6.3. Allocated Memory

Memory requirement is not very important when algorithms are implemented on modern computers; however, it will be important when mobile platforms with limited computational power are used, especially in real time. For this calculation, the memory function in MATLAB is used. Results are dependent on the computer hardware and the load on the computer.

## 3. Results

Table 1 presents results of the average F1 score in the 68 aECG records using different approaches. The combination of TS-FastICA yielded the highest F1 score with 92.61%, followed by JADE-TS-JADE and TS-JADE methods with 91.56% and 91.16%, respectively. The lowest F1 scores were found in EKF, JADE, FastICA and RobustICA, with 54.34%, 61.27%, 60.08%, and 59.60%, respectively. It should be noted that using these algorithms alone was not as effective as when they were combined with other algorithms. Once motion noise was added, the F1 score was reduced significantly. More specifically, the highest F1 score was below 90%, including TS-RobustICA, JADE-TS and JADE-TS-JADE. The lowest F1 was from EKF with 51.45 %.

In Table 2 the number of records that had a F1 score lower than 50% is illustrated, providing a comprehensive assessment about the reliability of each approach. Specifically, the use of a combination of different approaches resulted in the lowest number of records with an F1 score below 50%, while using individual approach increased the number of records with an F1 score below 50%, especially in EKF, with 38 out of 68 records. This is important, since many of the previous works that used EKF for fECG extraction illustrated their work’s result using a limited number of records. However, we applied EKF on all 75 recordings and although some extremely high F1 scores have been achieved, for 38 records an F1 score lower than 50% was achieved. Similarly, the data with added motion noise showed a higher number of recordings with an F1 score below 50% which is reasonable as motion noise may dominate fECG, which has lower amplitudes.

**Figure 6.**
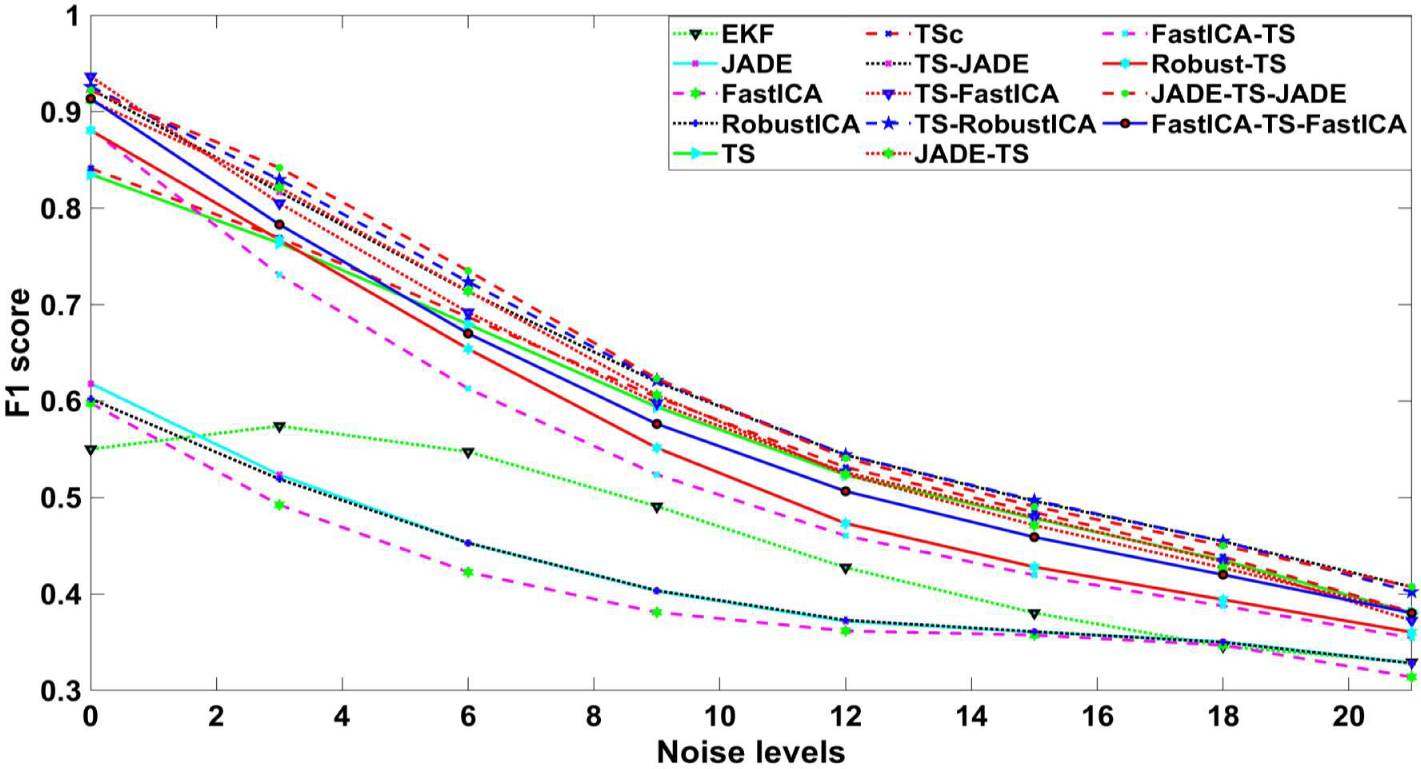
F1 comparison with different Gaussian noise levels.

The performance of studied approaches with noise-added data is described in Figure 6. It is obvious that, with higher noise levels, lower F1 was achieved. All approaches showed a linear curve for F1 score when increasing the noise level, except for EKF. The F1 score in EKF slightly increased in noise level 3, compared with that in noise level 0. It could be suggested that the noise added may superimpose the fECG signal, inadvertently easing the ability of this method to find peaks in fECG signal.

**Figure 7.**
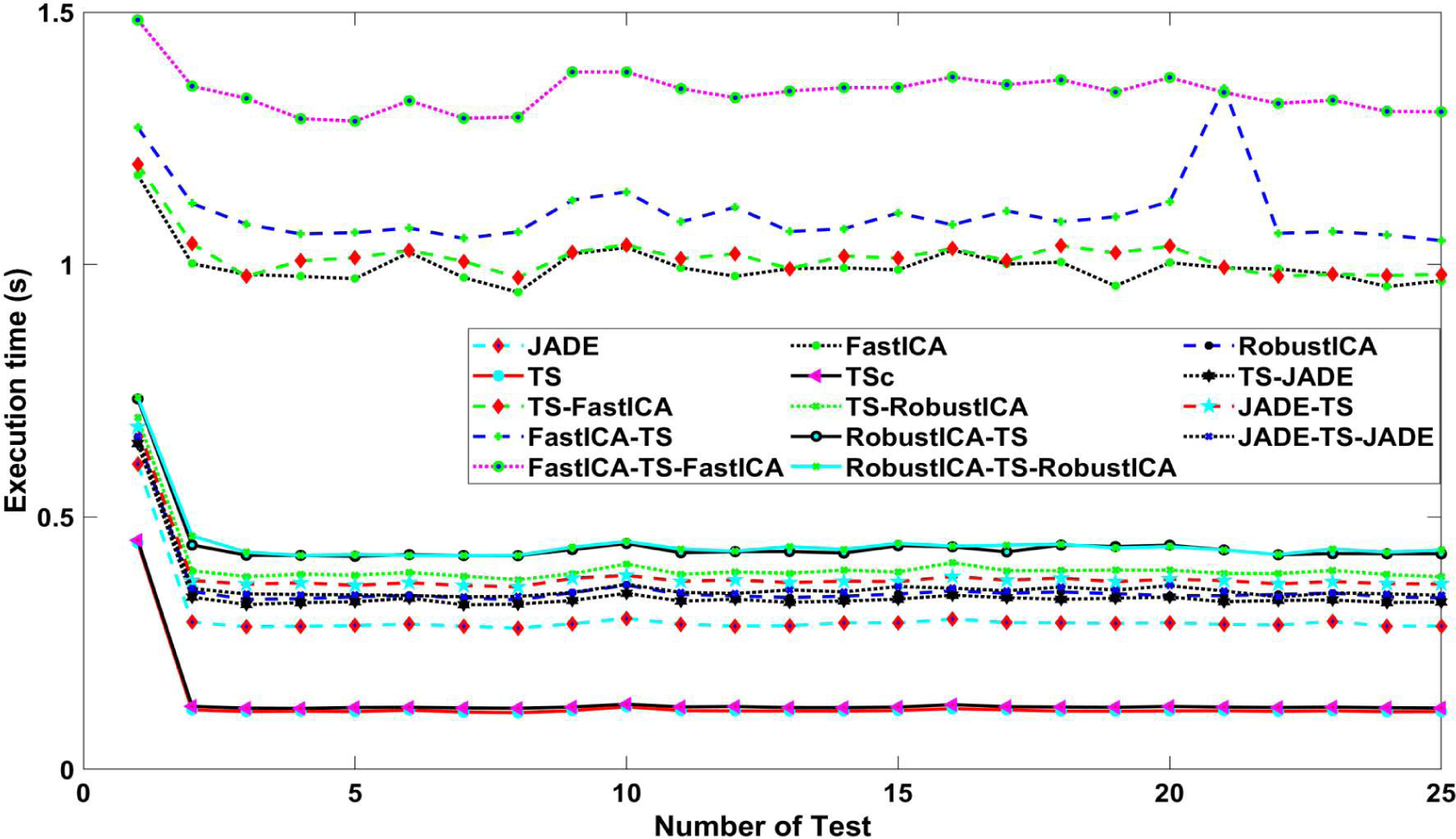
Time execution comparisons.

Table 3 shows the amount of memory used for each algorithm. The EKF method occupied the highest memory capacity with 2940 MB, while other algorithms showed memory below 1300 MB. Although a combination of different methods could provide higher performance, i.e., higher F1 score, it took higher amount of memory than running them individually. For both high-performance and low memory occupied case, JADE-TS-JADE would satisfy that criteria. Figure 7 depicts the time execution ran multiple times for each algorithm. In general, the time execution did not change significantly during each running. The approaches with FastICA method including, e.g., FastICA-TS-FastICA, TS-FastICA, FastICA-TS, FastICA, showed the highest time execution with nearly 1 s. Other approaches fluctuated within 0.75 s in first few running and stayed at 0.3 s after that. TS and TSc methods took the lowest time to finishing the program.

In EKF, we used a beat-fitter module that calculates *α*_*i*_ and *b*_*i*_ (*α*_*i*_ and *b*_*i*_ represent the amplitude and width of the Gaussian functions of the five PQRST waves). In this module, a number of kernels should be placed in critical points of the ECG. Both automatic and manual beat fitter modules were tested, but the manual module is more robust. However, for the manual one, calculating the computational time is impossible, since it ends when the user is done with choosing the kernels. On the other hand, in the automatic module based on the desired minimum error of the dynamic model, the computational time varies. The average computational time in automatic EKF is 21.7 s.

## 4. Discussion

We compare different fECG extraction algorithms via assessing five well-defined criteria: the raw data, the motion noise, the white noise, the execution time and the required memory. Using the original data, the F1 score widely varies, from 92.61% (TS-FastICA) to 54.34% (EKF). Algorithms with the same topology have relatively close F1 scores. The results of TS algorithms are fairly consistent, and the same thing happened to ICAs. TS algorithms give better results than pure ICA algorithms and when we combine them, we get a higher F1 score. With motion noise added, algorithms such as EKF and ICA have an accuracy reduced by only 1% to 3%. However, for TS algorithms, F1-score was reduced by more than 11%. Overall, JADE-TS-JADE has the best performance and has the highest accuracy. However, RobustICA-TS shows the lowest accuracy reduction after adding motion noise. In other words, RobustICA-TS is more robust. According to the results presented in Table 2, JADE-TS-JADE has the least number of records with F1 scores lower than 50% and EKF has the most F1 scores lower than 50% indicating the reliability and consistency of methods. Although the result of JADE-TS-JADE is the most reliable, the EKF algorithm only requires one channel for data acquisition and fECG extraction, while other methods need four channels of aECG. This indicates that, with EKF, the physical device would be smaller and more unobtrusive.

Figure 6 illustrates the performance of algorithms in the presence of white noise with different amplitudes. In high SNR (low white noise), TS-FastICA has the best performance while in low SNR (high white noise), TS-JADE works better. Note that in very low SNR, data were completely corrupted, and the performance of different methods was found similar. The next criterion is allocated memory for fECG extraction. Table 3 presents the required memory for these methods. The required memory for all methods was less than 1300 MB, except EKF. EKF needs much more memory for fECG extraction. Since for home-based monitoring, algorithms run on microprocessors, the required memory could be important.

Figure 7 shows the computational time for these methods (excluding EKF). Execution time, which is the time it takes for the mentioned computer to process one of the 75 recordings (60 s) from the dataset is vastly different. TS algorithms need the least time, 0.128 s, while that of FastICA-TS-FastICA is 1.5 s, which is the slowest running algorithm. We could see that four algorithms containing FastICA are very slow. The reason is that, in some cases, the FastICA algorithm did not converge, depending on several initial values, while other algorithms are quite stable. If we remove those cases, the execution time of these algorithms will be approximately that of the same topology algorithms.

We noticed that the execution time of TS and TSc is shortest. The execution time of algorithms containing FastICA and RobustICA (such as FastICA-TS and RobustICA-TS; TS-FastICA and TS-RobustICA etc.) is nearly equal. Meanwhile, if we replace FastICA or RobustICA in those algorithms with JADE, it would take less time. In addition, we observed that JADE-TS runs slower than JADE-TS-JADE. The reason for this is that JADE-TS takes more time in the block fQRS detection and selection. The same thing happens with FastICA-TS and FastICA-TS-FastICA pairs. However, the advent of high-speed processors has made this factor less important.

## 5. Conclusions

We have carried out a comprehensive study of different methods for fECG extraction. Accuracy, noise robustness and simplicity were utilized as criteria to compare, which holds promise to enable a home-based monitoring system for the unborn children as well as the expectant moms. In such scenarios, the signal most likely interfered with motion artifacts; therefore, the performance of an algorithm in data with motion is preferred. In this case, JADE-TS-JADE showed the best performance in terms of the aforementioned criteria. The EKF has the weakest performance, especially with noise added; however, it requires only one channel for fECG extraction, thus bringing compactness in manufacturing, and thus possibly being widely accepted by users. In future work, our team will focus on cloud-based analytics of fHR/fECG and mECG for promoting maternity care, as well as novel algorithms to reliably extract fECG with full features of P waves, QRS complexes and T waves for the early detection of congenital heart disease and critical events during pregnancy.

## Figures and Tables

**Table 1.** Average F1 score (%) with different methods for all records.

Method	Without Motion Noise	With Motion Noise
TS-FastICA	92.61	85.02
JADE-TS-JADE	91.56	85.43
TS-JADE	91.16	82.35
TS-RobustICA	90.71	80.63
JADE-TS	90.57	85.10
RobustlCA-TS-RobustICA	89.29	82.67
RobustlCA-TS	87.43	83.21
FastICA-TS-FastICA	87.07	82.47
TSc	83.12	70.64
FastICA-TS	82.96	77.94
TS	82.65	71.02
JADE	61.27	59.81
FastICA	60.08	59.38
RobustICA	59.60	58.74
EKF	54.34	51.45

**Table 2.** Number of records out of 68 datasets with F1 scores less than 50%.

Method	Without Motion Noise	With Motion Noise
EKF	38	40
RobustICA	28	28
FastICA	22	27
JADE	18	19
TSc	10	17
TS	10	17
FastICA-TS	6	9
FastICA-TS-FastICA	5	5
RobustICA-TS	5	5
RobustICA-TS-RobustICA	2	5
TS-RobustICA	2	8
TS-JADE	1	7
TS-FastICA	1	4
JADE-TS-JADE	1	1
JAD E-TS	0	3

**Table 3.** Required memory for different methods.

Method	Required Memory (MB)
EKF	2940
JADE-TS	1222
TS	1220
TS-FastICA	1211
TS-RobustICA	1210
FastICA-TS	1206
TSc	1205
TS-JADE	1204
RobustICA-TS-RobustICA	1202
RobustICA-TS	1199
FastICA-TS-FastICA	1199
RobustICA	1199
JADE-TS-JADE	1192
FastICA	1183
JADE	1175
